# Development of Induction Heating System Ensuring Increased Heating Efficiency of the Charge Material in a Forging

**DOI:** 10.3390/ma15041516

**Published:** 2022-02-17

**Authors:** Marek Hawryluk, Marcin Rychlik, Michał Pietrzak, Piotr Górski, Jan Marzec

**Affiliations:** 1 Department of Metal Forming, Welding and Metrology, Wroclaw University of Science and Technology, 50-370 Wroclaw, Poland; michalpietrzak99@gmail.com (M.P.); piotr.gorski@pwr.edu.pl (P.G.); jan.marzec@pwr.edu.pl (J.M.); 2 Kuźnia Jawor S.A., 59-400 Jawor, Poland; marcinrychlik@kuznia.com.pl

**Keywords:** induction heating, charge material, simulation and modeling, distribution of temperature

## Abstract

This study performs a complex analysis and review of the currently applied methods of inductively heating the charge material in hot die forging processes, as well as elaborates and verifies a more effective heating method. On this basis, a device for inductive heating using variable frequency inductors was designed and constructed, which made it possible to reduce the scale and decarburization with respect to the heater used so far. In the first place, the temperature distributions in the heater in the function of time were modeled with the use of the CEDRAT FLUX software. The aim of the research was to analyze the temperature gradient and value diversification on the surface and in the material core, as well as to determine the process stability. The following stage was designing and constructing a heater with an automatic system of loading and positioning of the charge on the exit, as well as with a possibility of working in a fully automated system adjusted to the work center. The last stage of investigations was the verification of the elaborated effective heating method on the basis of a short production series and a continuous work for the period of 8 h, both in the quantitative and qualitative aspect (reduced oxidation and decarburization as well as a gradient between the core and the surface). The obtained results confirm the effectiveness of the proposed solution referring to heating the charge material, especially in the aspect of stability and repeatability of the process, as well as a significant reduction in oxidation and decarburization of the material surface.

## 1. Introduction

The processes of heating the charge material in a die forging process are constantly improved, yet they still pose a challenge scientifically, technologically, and economically. Currently, one of the most common methods of heating the charge material to a given temperature for the die forging and extrusion processes is induction heating, which has a great competitive advantage over other alternative methods of heating the charge [1,2]. The main problem to be solved during the heating of the charge is undoubtedly oxidation and decarburization of the charge surface, which has consequences on the quality of the produced forging. The subject literature states that, depending on the heating parameters, even 3% mass volume of steel can be turned into scale during heating, thus going to waste, and over 1% constitutes decarburization. A significant percentage of the annual production of steel elements in Europe is questioned due to too much scale and carburization [3]. Considering the huge scale of steel forging production, we can estimate the amount of wasted material and the importance of the loss of surface layer properties related to those defects [4]. Reduced properties are especially negative for carbon steels, whose properties are based on the content of that element. Oxidation and decarburization of steel take place simultaneously during heating, mainly in an oxidating atmosphere [5]. Decarburization at elevated temperatures has led to a continuous diffusion of carbon from the areas close to the surface towards that surface, which has resulted in decarburization of the materials surface layer. An effect of decarburization is lowered hardness in this layer, and thus also a drop of the expected performance parameters [6]. Sometimes, the heat process parameters are selected in such a way so that the steel oxidation can take place more rapidly than the carbon diffusion, and thus also the decarburization [7]. A popular solution is the use of protective atmospheres with a reduced content of reactive oxygen. The protective atmosphere is created by protective gases, such as methane, ethane, propane, argon, hydrogen, helium, nitrogen, or alternatively, vacuum [8]. Unfortunately, introducing a protective atmosphere is not always possible due to the limitations resulting from the specificity of some processes realized in open space, but mostly for economic reasons, which is the case of large production series of forgings for the automotive industry. In such cases, protective coatings can be applied, whose task is to separate the surface from the reactive atmosphere of oxygen. It can be pastes or liquids with various contents used to cover the material surface [9]. The most popular coating techniques are submerging, application with a brush, spraying, and lubrication. Some of them guarantee a more precise coating of the surface, while others ensure a thin layer, thus reducing the consumption of the protective agent. The main problem with the use of coatings is the temperature at which the steel is heated before the forging process; in extreme cases, the charge reaches over 1300 °C, which significantly limits the spectrum of materials that can be used for that purpose. One of the popular groups of protective coatings is graphite-based media. The advantages of water graphite suspensions include excellent lubrication properties, reduced tool wear during forging, a wide range of possible graphite grain sizes (up to 50 μm), well-coated products, with them being easy to dilute, economical, free of ammonia [10].

Besides the mentioned method of applying protective atmospheres and coatings, in order to reduce the amount of scale and decarburization, we should shorten the time of exposure of the charge to the effect of heat, as well as optimize the time and key parameters of heating, and also accelerate the forging process, e.g., through its automatization [11]. Currently, the main direction of such measures is the use of induction heating of the charge material, owing to high efficiency, easy implementation, and robotization, as well as relatively low costs, with the consideration of ensuring the best quality of the heated charge, characterizing in low decarburization and scale [12,13]. At the same time, it is being constantly optimized with respect to the first implementations of such a heating method (in reference to the alternative, resistance heating) through reducing the energy consumption, increasing the efficiency and the quality of the heated material [14,15]. The main problem existing in the construction of devices at the beginning of their implementation was the heating technique itself [16,17,18] as well as the temperature stabilization with a linear course, which unfortunately caused significant decarburization and scaling of the external surface of the material, especially in the case of a steel charge with a high carbon content [19]. Additionally, we can differentiate between sections of constant and varying frequency inductors. In the case of the latter (more recent solutions), there is the possibility to select the proper frequencies of alternate current, which makes it possible to heat charges with larger diameter scopes (a more universal yet more expensive solution), with respect to constant frequency inductors, which are used for a few diameters of bars with similar diameters [20,21]. In turn, in older solutions, where the heaters were not divided into sections, the changes in the heating speed, aimed at obtaining the assumed temperature and working cycle, are realized through a change in the distance between the consecutive links of the coil [22]. In the case of induction heating, an important aspect of the heating parameter selection, besides the construction of the device itself, is ensuring the proper temperature distribution in the whole volume of the charge. Heterogeneity of the temperature field distribution can cause the so-called “skin” effect, which is an improper course of eddy currents as a result of improper voltage-frequency parameters, which causes e.g., a higher temperature right beneath the surface and a lower one on the surface and in the core [23]. The most popular control methods are a double pyrometric control and a thermovision measurement, where the devices are installed on the end of the heating zone, and the measurement of the characteristics takes place right after the material is removed from it [24]. The recorders read out the extreme values on the length of the examined material and, through communication with the machine, separate the underheated charge—suitable for another use—from the overheated one, which has to be utilized due to possible internal defects [25,26,27].

An important aspect of adjusting the heater to the production conditions is the adjusting of the heating time while maintaining the assumed parameters and homogeneity of temperature distribution to the production cycle and equalizing the temperature in the whole volume of the charge through its annealing at the last stage of heating. The most advanced technologies enable even greater universality to apply varying frequency inductors [28,29]. Modern solutions have made it possible to equip the device with automatic pushers of the charge wound around an electrically powered barrel. In the case of the end of production, the links are automatically unwound until they reach the position on the end of the last inductor, from where the last charge material is transferred for die forging [30]. In the case of the automatization of the whole forging process, one important aspect affecting the process implementation and the number of staff needed to operate the device is also the method of loading the charge material, which further has to be properly positioned before being pushed to the heating zone. In the case of constructing the whole automated forging bay, it is crucial to minimize the human factor in the sense of ensuring stability and repeatability of the process, and the works connected with loading and positioning of the material in a specific way are typical steps, which can be performed by a robot or another executive system. The current trend observed not only in the case of heating devices is introducing universality through their module construction, which enables a quick reconstruction or replacement of one of the modules and the next initiation of different productions. In modern solutions, the devices are built-in sectors, where each segment is powered from a source located in a cabinet under the inductor. Owing to such a solution, in the case of increasing the production capacity resulting from a development of the line or its automatization, there is a possibility to purchase additional equipment instead of ordering a whole new heating device. It should be mentioned that to develop solutions for induction heating, a number of engineering and IT tools are often used, mainly focused on numerical modeling of the heating process [31,32,33]. By using FEM-based computing packages and other tools based on a virtual experiment, it is possible with high accuracy and in a very short time to develop and verify the developed solutions and concepts [34,35,36].

The aim of the study is to analyze the effect of the developed effective method of induction heating on the stability and repeatability of the process, as well as the amount of decarburization and oxidation of the heated charge materials surface.

## 2. Materials and Methods

For the development of an effective and proper technique of heating the charge material (assigned for forgings for motor car steering system components), a series of tests and analyzes were performed with the consideration of the solutions available on the market and at other forges. The main aim of the studies is to obtain a repeatable and stable temperature distribution as well as the possibility to reduce the size of decarburization and oxidation (scale) of the charge during the series production of forgings from C45 steel in respect of the currently applied solution. So far, the applied heating system has been based on two-section constant frequency inductors, which makes for an impossible smooth regulation or fixing of the key time-current parameters. Additionally, during the design of a new, more effective charge heating system, the possibility of adjusting such a device to operating in an automated forging bay was taken into consideration. Within the performed studies and analyzes, the possibility of automatization of the material loading, the temperature distribution in the heating section, and the manner of positioning the material to be collected by the robot was verified. The investigations were conducted for a double charge length (due to the consideration of forging in a multiplied-double system in the future) in respect to the solution applied so far, where one forging has been produced from one heated charge. The main tests were divided into four research tasks, including:

- Modeling and analysis of the temperature distribution in the heater in order to obtain the assumed charge temperature parameters (in the core and on the surface);
- Development of a heater construction with the consideration of automatization and the positioning of the heated material;
- Verification of the developed solution in the aspect of ensuring the stability of the charge temperature parameters and repeatability of the heating process;
- Analysis of the effect of the elaborated heating system on the quality of the charge material.

In order to solve the above issues and problems, the following information-engineering tools were used: for CAD designing (Catia V5, DraftSight 2020); for numerical modeling FEM (Qform VX9.09); for temperature change modeling (Cedrat Flux 2D/3D); for the measurements of the temperature field changes and distribution, a thermovision camera was used (FLIR T840, FLIR Systems, Inc. Wilsonville, OR, USA) as well as a pyrometer with a thermocouple type K (Testo-845, Testo Poland, Pruszkow, Poland), and also other classic measuring tools.

## 3. Tests and Results Discussion

The investigation was divided into four main research stages. Research work planned in this way and then carried out allowed for the development of an induction heating system, ensuring increased heating efficiency as well as reduced scale and decarburization of the charge material in a forging, which was additionally verified in industrial conditions.

### 3.1. Modeling and Analysis of the Temperature Distribution in the Heater to Ensure Stabilization of Charge Temperature Parameters

As the first stage of research, after determining the preliminary concept of the solution with the use of the Cedrat Flux 2D/3D software [37], numerical modeling was performed for the temperature distribution in the transition zones and the assumed inductor parameters in the elaborated induction heater. The heating temperature is an especially important aspect in reference to the quality parameters and mechanical properties of the forgings’ constituting components for automotive production, where the expectations of the buyers are much higher than in other branches of industry. At present, the technologies are based on devices equipped with sector inductors, with the possibility to control the voltage and the shift speed in the heating section, which makes the gradient on each section changeable [38,39]. The most common solution is dividing the heating section of the induction heater into three or more parts, depending on the size of the device [40,41]. In the case of the most commonly used low- and medium-carbon steels for forgings, the first, longest part is responsible for a temperature increase to 550–600 °C as well as for leveling the temperature between the core and the external part of the material. The central section (inductor) is responsible for an increase to about 850–900 °C, with temperature leveling on the cross-section of the charge material. In the last section, in which the operation of the field is much higher than in the previous inductors, rapid annealing up to the desired forging temperatures values is realized (usually 1150–1250 °C) due to the limitation in the grain growth and decarburization of the surface, after which the material is removed from the heating area and transported to the forging stand [42,43,44]. Shortening the annealing time in the values required for the process will cause a reduction of surface decarburization and weight of the surface scale, thus increasing the quality of the ready product [45,46,47].

The aim of the studies was to analyze the temperature gradient, the value diversification on the surface and in the core, and the determination of the process stability. On the basis of the preliminary literature analyzes [48,49] and our own experiences, a varying frequency solution was selected. The material heating zone was divided into three sections, with the possibility to regulate the power, depending on the bay’s efficiency and the weight of the produced forging. During production with reduced efficiency (longer cycle time), as in the case of products with a yoked shape, the reduction of the shift speed is correlated with a reduction of the device’s heating power. The inductors were designed with the concept of varying frequency heating with diversified power of the particular sections, with the assumption of minimized oxidation and decarburization of the material. The first inductor (section) is responsible for annealing the charges up to the scope of 600–700 °C, while the second one causes an increase to 1000–1050 °C and stabilization of temperature towards the core, whereas, in the last section, we observe an increase of temperature to the one expected in the process, oscillating within the scope of 1150–1220 °C.

**Figure 1:**
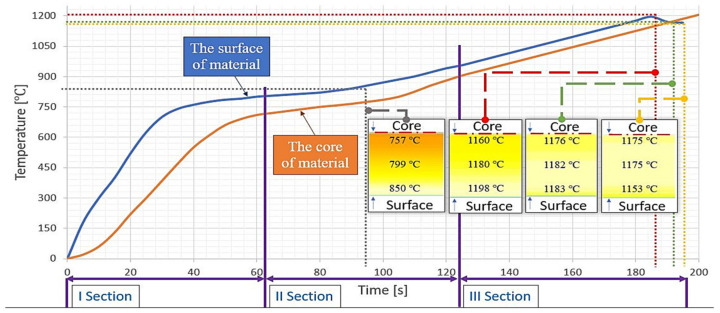
Temperature on the surface and in the core of the heated material in the function of time.

The investigations began with a simulation of heating for the assumed temperature parameters in order to obtain a specific heating time and a similar temperature in the core and on the surface after the charge left the heater (1200 °C). In the case of the material selected for the tests, with the dimensions of Ø35 × 132 mm, an inductor with the working space of Ø40 mm (coil diameter 55 mm) was applied (simulated), and with the calculated heating current of 2187 A, the effect of which was efficiency at the level of 705 kg/h, and the expected output temperature was 1200 °C, with the heating frequency of about 1500 Hz. The assumptions assume a copper coil with a rectangular cross-section with dimensions of 10 × 23 mm (width × height) used in each of the three sections with a length of 600 mm each. Detailed simulation studies followed by analyzes were performed consecutively for heating in the middle of the central inductor at the moment of exit from the heating zone, as well as 5 and 10 s after the material left the inductor. In order to perform the simulation correctly, it is necessary to recreate the parameters of a given induction heating process. TET4 and HEX elements were used to implement the geometry necessary to simulate the process, and the parameters were set in accordance with the characteristics of the material. TET elements of the mesh were used to model the geometry volume, while HEX elements were used to model the surface area of the detail, where the eddy currents share is significant. In the case of the induction heating simulation, both electromagnetic and thermal issues were considered. The material used for the charge (C45 steel) was selected from the program material database. For electromagnetic parameters, the magnetic properties of steel in the form of a curve B (H) as a function of temperature were taken into account, where B is the magnetic induction vector (T), H is the vector of the magnetic field intensity (A/m). The value of magnetic saturation was assumed at the level of 2 T (to the Curie point 760 °C), while the energy of the phase transition (1.2 (GJ/m^3^)). The parameters were selected from the material database of the program. Additionally, during the simulation, the thermal conductivity of steel, resistivity, and heat capacity was considered. The above values change as a function of temperature, which was considered in the simulation. The initial thermal conductivity was assumed to be the value (47 [W/(mK)]) with the change of value as a function of temperature (–0.00025 (1/°C)), heat capacity (3.9 [MJ / (m^3^ × °C)]), and the steel resistivity (250 (nΩ/m)). For thermal parameters, the following were considered. The heat transfer coefficients were assumed to be (20 [W/(m^2^ × °C)]) for convection and (0.5 [W/(m^2^ × °C^4^)]) for radiation. The ambient temperature was assumed to be 20 °C. The simulation properties were assumed to be variable as a function of temperature; isotropic magnetization described by the B (H) curve taking into account the nonlinear behavior of the permeability coefficient and the maximum distribution of magnetic saturation with exponential distribution, isotropic resistance with linear distribution, isotropic thermal conductivity with linear distribution, and heat capacity with Gaussian distribution. The simulation results, together with the course of the heating process, have been presented in Figure 1. Due to the fact that the speed at which the material is pushed in the assumed solution was predetermined as constant, and each section is equally long, the time of presence in each segment equals about 67 s. In the first section (Figure 1), according to the assumptions, pre-annealing to about 700 °C in the core and 800 °C on the surface takes place. Initially, the temperature increases equal to 25 °C/s, and after reaching 750 °C on the surface, the increase is reduced to 2 °C/second (at the end of the inductor). The second segment enables heating to the value of 950 °C outside and 880 °C on the material’s axis. The temperature increase initially equals 2°C/s (at the beginning of the inductor), with an increase to 5 °C/s 15 s after the material is transferred through the incubator. A lowered temperature at the end of the first and beginning of the second section positively affects the leveling of temperatures between the surface and the core and annealing at 800 °C should not cause significant decarburization, which is in agreement with the material’s decarburization curve [50].

The heated steel bars (charge) at the moment of exit from the last incubator reach 1176 °C inside and 1198 °C on the examined surface, with an increase of 5 °C/s, similar to the second section. After the exit from the heating zone, cooling of the external surface takes place and a slight temperature increase is observed, caused by conduction, and leading to leveling the balance at the cross-section of the charge. After about 5 s from leaving the heating zone, temperature leveling occurs, where the difference between the external surface and the core does not exceed 7 °C, and the material surface maintains the value of 1183 °C. The following 5 s of waiting for the collection causes a significant reduction of temperature on the surface to the level of 1153 °C when it is maintained at 1175 °C, due to which fact both the scatter and the surface decarburization (resulting from the time of holding at the forging temperature) demonstrate worse conditions than in the previous case (after the first 5 s). Moreover, considering the robotization of the developed heating system, the collection by the robot should take place after about 2–3 s from the moment of leaving the heating zone, and the placement for forging-after the following 2 s, where, according to the simulation data, the distribution is the closest to ideal. Based on the conducted investigations, calibration was also performed on the device eliminating the charge when the time of waiting for the collection is too long, i.e., 8 s, in order not to produce from a material with a significant scatter of temperature in the cross-section.

### 3.2. Designing a Heater Construction with the Consideration of Automatization and Positioning of the Heated Material

**Figure 2:**
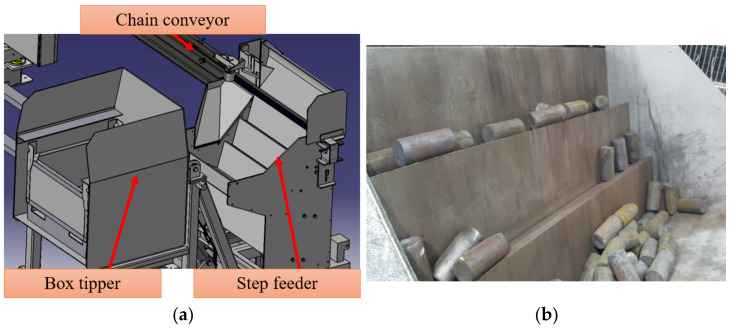
View of: (**a**) the model of a feeder with a tippler, (**b**) the board feeder of the heater.

The following stage of research was investigations connected with automatization of the heater, among others, in order to reduce the staff to the necessary minimum required to operate the device, as well as to provide the possibility for the device to work in the production bay. The technological line was designed in such a way that it would begin with a box tippler (Figure 2b), with bars cut into the desired length. Regardless of the manufactured product, a container with a gross mass not exceeding 2000 kg is introduced by means of a forklift truck to the turnover station. After the closing of the shields protecting the work of the hydraulic engine powered mechanism, the charge is thrown over to the chamber of a stepped feeder. Next, the three-level mechanism lifts the randomly positioned material with interchangeable boards, lifting it onto a chain feeder leading towards the heating element (Figure 2a). After the leaving of the lifting zone, there comes the eliminator element, which, through a mechanical position control, casts away the badly positioned bars again into the feeder chamber, so that only the properly arranged charge material can undergo the forging process. The non-heated cast-away material is lifted again through a board feeder until it reaches the proper position, enabling its transport to the heating section. In the case of improperly cut material, it is eliminated at the stage of entering the process or during the length measurement (with the use of an encoder) before being introduced into the heating section. After leaving the chain conveyor, the bars are transferred to the mechanism pushing them into the inductor, where the material is transported in a pushing manner. The guiding in the inductor is ensured by cooling pipes installed around the inductor, which are simultaneously responsible for cooling the lining, thus, preventing machine failure.

Additionally, on the end of the inductor, a double pyrometric control has been installed, which performs surface verification in two planes on the whole material length. The aim of the measurement is to cast away the underheated material, which can cause forging machine failure or lower the quality of the ready product, as well as the one which is overheated beyond the accepted scope; unacceptable for metallographic reasons on this basis. For the heating of the charge material, an induction heater with replaceable inductors and mechanical guide regulation was selected, with the guides positioned depending on the diameter and length of the charge bars.

Moreover, during the designing works to adjust the heater to the whole production bay, the device was equipped with modern automatization systems for charge material preparation, through the introduction of, e.g.:

- Conveyors connecting the feeding of the material with the heating section, where, during the movement, the charge material in the form of cut bars are appropriately oriented so that each bar is axially introduced into the inductor;
- A system of introduction into the inductor equipped with automated control of material presence, in order to counteract production shutdowns caused by a lack of charges;
- A system of temperature segregation equipped with the option of measurement, as well as an algorithm enabling material transfer to the forging section or its elimination (in the case of defects).

The next step of research in this stage was an elaboration of a charge material positioning technique which would make it possible to maintain a stable collection point with the option of adjustment to the applied charge material sizes, which is the adjustment of the heater’s end (device) to the possibility of a stable collection by the grippers in the robotized process. An additional aspect required at this stage is the recognition of the number of charge material bars in the case of welding in the heater and a drop of two heated bars at the same time, as well as the time limitation enabling elimination of the material that has been waited for collection for too long. The issue was solved by design and research works, as a result of which, based on many considered concepts and analyzes, it was decided to apply regulated planking. The setting option was designed in a solution not requiring specialized tools, in such a way so that the operator could adjust the width of the downpipe to the required value, by referring to the designations on the machine. The end of the heater on the side of the robot was equipped with a screw mechanism with a scale, which enables the regulation of the collection position for the robot. Additionally, the collection point was equipped with optical sensors on a regulated frame with the element of setting to the length of the produced material, which enabled control of the number of bars waiting after the heating. In the case of welding or when the material is waiting by cooling down after the above set time, the lower lock is powered by a pneumatic servo motor in order to cast away the charge into a bin located below the chutes, thus ensuring the safety of the forging press. It should be emphasized that the developed solution additionally eliminates the phenomenon of material welding during temperature increase (through the use of an element separating the consecutive bars—charge), owing to which the risk of placing a larger number of bonded charges in the press window is lower, as well as there being no possibility of forging from a cooled material.

### 3.3. Verification of Heating Process Stability

**Figure 3:**
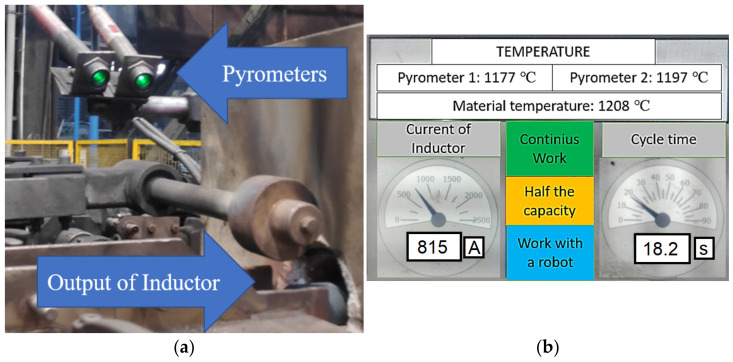
Images of: (**a**) the pyrometers installed on the heater, (**b**) the HMI temperature measurement panel.

The results obtained in the simulation were verified under industrial conditions. To that end, based on the performed numerical simulations of the temperature changes, the component elements of the heater were selected and a complete device was built, which was followed by verifications tests. The verification was performed through a temperature measurement of the charge on the surface by means of two pyrometers positioned on one side of the heated material at a distance of 40 mm. The applied pyrometers (Figure 3a) realize a continuous measurement of temperature, where the driver, analyzing the data, displays the highest examined value on the screen (Figure 3b), in order to exclude the overheated material, which, in such a situation is eliminated due to microstructural changes. Exceeding both the upper and lower limit of the assumed temperature causes automatic segregation after the material leaves the heating section, whereby the technology accepts a situation when an underheated charge can be used again after being cleaned off the scale. The tests were conducted on a production material, steel C45, for which, in the case of the produced forging, exceeding 1225 °C is unacceptable. For these reasons, the proper values were set at the level of 1200 ± 25 °C.

**Figure 4:**
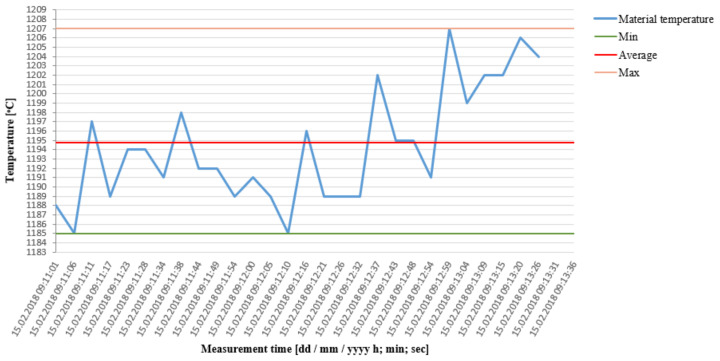
Temperate values for the consecutive 30 charge materials during a 15-min trial.

Next, two trials with different test times were performed. The first trial lasted 15 consecutive minutes (during a prolonged work of the device), where the deviation from the assumed temperature of the successive charges was verified. In turn, the second trial, lasting 8 production hours, for which the measurement was realized at constant intervals, was conducted in order to examine the stability of the process in a longer time period of the device’s operation. In the research trial lasting 15 min, 165 bars were heated to the assumed temperature. Figure 4 shows the results for the consecutive 30 items (out of 165), recorded during the middle part of the trial. As a result of the performed analyzes, the maximal scatter of 22 °C and the mean value of temperature on the charge surface at the level of 1195 °C were obtained. The maximal deviation from the assumed nominal temperature (1200 °C) was estimated as 15 °C. From the heated 165 preforms, during the temperate segregation, 11 items were cast away at the beginning of the process, before the set working parameters were reached (during pre-annealing of the charge).

Besides the material used to reach the working parameters during the tests, no other charges with a temperature deviation beyond the acceptable tolerance were observed. The obtained results were deemed as correct and maintained within the acceptable deviation.

**Figure 5:**
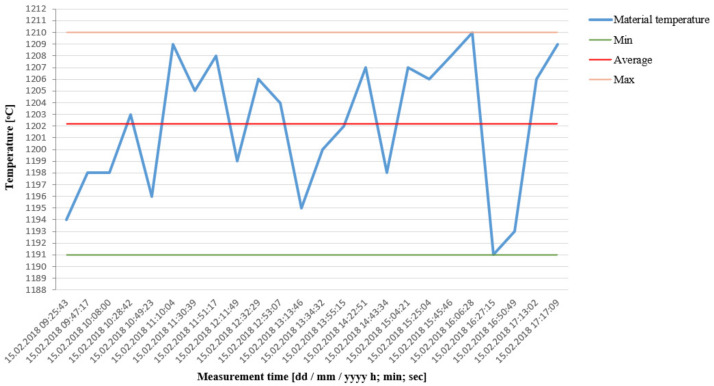
Values of periodical temperature measurements of 24 random charge materials during the 8-h trial.

The other test trial was conducted in the time of 8 h, during which the device worked without stopping. Besides a continuous pyrometric control every 20 min, the results were verified by means of a manual device (pyrometer) in order to perform additional control measurements. The results of the industrial trial have been shown in Figure 5, where an analysis was made of 24 samples separated from each other time-wise.

**Figure 6:**
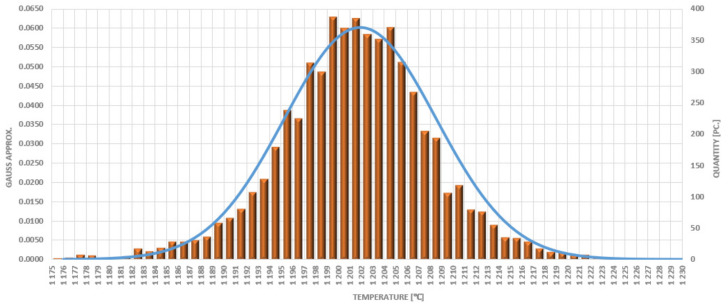
Distribution of the occurrence of particular temperatures of the input material during the 8-h trial.

In the trial, only six preforms were eliminated, due to too high a temperature, as well as those which were used to reach the working conditions at the start-up and during the particular shutdowns (total of 59 charges). For this reason, the operation of the device was deemed in accordance with the production assumptions and the requirements of the automotive industry. During the whole test, 5.764 charges were heated, out of which 59 items were cast away due to underheating during the start-up of the device at the beginning and during production shutdowns as well as 6 due to overheating. Figure 6 shows a collective compilation of the occurrences of particular temperature values together with the number of occurrences in the process.

Statistically, the distribution is close to normal, with a light shift towards elevated temperatures. The most occurrences (350–380) were within the scope of 1200 °C to 1206 °C. The reject rate was determined at the level of 1.02%, of which 0.1% was due to overheating. In total, the results of the trial were established as positive. During the whole trial, the average equaled 1199 °C, the minimum was 1176 °C, and the maximum at 1230 °C (1 item).

**Figure 7:**
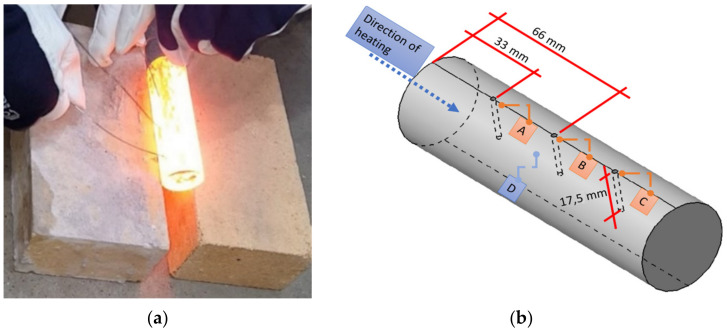
View of: (**a**) a temperature measurement with the use of thermocouples, (**b**) schematics of the material prepared for the tests.

Simultaneously, temperature measurements were made in two parallel ways, through measurement by means of a thermovision camera at the moment when the material leaves the heater, and control was performed with thermocouples after the drop onto the collection point. To that end, on each of 10 selected samples, 3 openings were drilled with the diameter of 2 mm and depth of 17.5 mm, reaching the material core Ø35 mm, situated in the center of the length (66 mm from the end, point B) as well as 25% from the ends of the materials (33 mm, points A, C), which has been shown in Figure 7.

The temperature measurements were performed simultaneously for all the measurement areas with the use of thermocouples and a recorder of results. Additionally, in order to evaluate the temperature difference between the surface and the core, an additional thermocouple was used, applied onto the surface in the central part of the charge. To ensure proper and stable measurement results, the examined samples were arranged interchangeably with the charge material (every third charge), where the measurement results were read out after 5 s from the moment of exit of the drilled bars from the heating section. The data from the trial have been compiled in Table 1.

**Figure 8:**
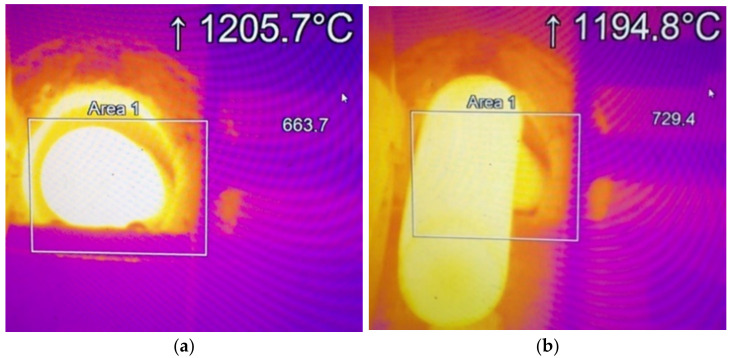
Thermovision camera measurement: (**a**) at the moment of exit from the heating section, (**b**) after the exit from the section.

Based on the performed tests, it was established that the maximal difference between the core and the surface of the material equaled 10 °C, and the minimal one at 5 °C. In most cases, a slight drop of temperature occurred in the core, with a tendency for a lower value on the side which had first left the heating section (maximal difference 5 °C). It was established that it was connected with a bigger heat loss on the surface of the charge, caused by a longer cooling time at ambient temperature. In turn, the temperature examinations with the use of a thermovision camera were conducted in order to examine the temperature changes on the surface between the front and back part of the charge at the moment of exit from the inductor. The trials were performed on 10 consecutive charge materials. The recorded temperature scatter reached its maximal value of 11°C (Figure 8), and the minimal one at 8 °C, which confirmed the tendency for a temperature drop from the tests with the use of thermocouples.

The performed analysis made it possible to confirm the results obtained in the tests with the use of numerical modeling in respect to the industrial conditions, as well as confirm the stability and repeatability of the process during its implementation.

### 3.4. Analysis of the Effect of Heating on the Charge Material Quality

**Figure 9:**
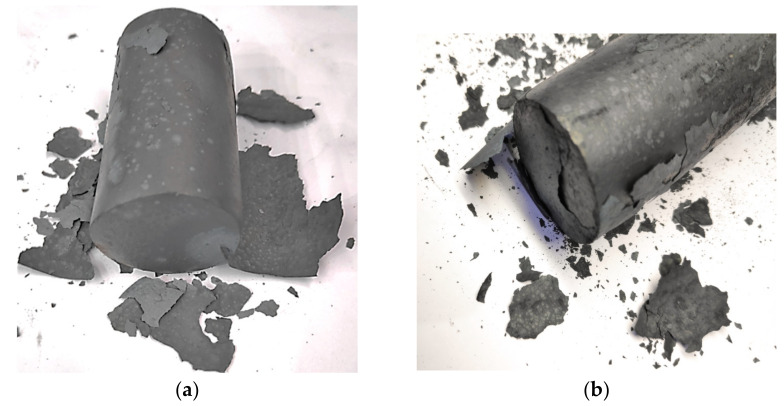
The shape of the scale after material heating: (**a**) during the manual process, (**b**) in the newly developed heating system.

**Figure 10:**
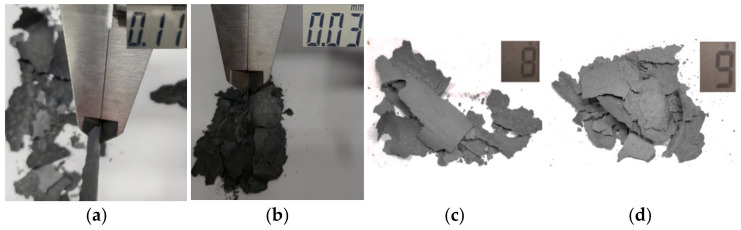
Thickness and mass of the scale after material heating: (**a**) thickness during the manual process, (**b**) thickness after the newly developed heating system, (**c**) mass during the manual process, (**d**) mass after the newly developed heating system (is double longer of charge material due to forging in a double system).

In order to verify the developed heating system in respect to quality, the disadvantageous effect of scale and decarburization on the charge surface quality was examined and analyzed. To verify the relations in reference to the elaborated (new) solution as well as the one applied so far, tests were performed in the scope of scale weight on the material surface, its type, and the microstructure of the charge material (Figure 9 and Figure 10).

In the technology applied so far, the charge material used for forging with the dimensions Ø35 × 53 mm, the surface area of 7750 mm^2,^ and the mass of 401 g is heated in a heater of an older type. The performed examination showed scale in its surface combined form (Figure 9a) with the thickness of 0.09–0.13 mm (Figure 10a). Next, the scale was removed, both in the newly developed solution and the one applied so far, in order to estimate the changes in mass as a result of surface oxidation, where the average of 8 g of material was obtained (Figure 10c), which constituted 2% of the total mass of the charge. The trial was repeated a few times, and the weight and thickness differences did not exceed 5% (0.4 g). In turn, in the case of the charge material for yoked forgings in a double system, heated in the newly developed device with the diameter of 35 mm, length of 132 mm, the surface area of 16, 438 mm^2^ and a mass of 998 g, scale in its fine loose form was observed (Figure 9b). The measurement showed a layer thickness of 0.02–0.04 mm (Figure 10b). An attempt at removing the scale was made, where 9 g of material was obtained (Figure 10d), which constitutes 0.9% of the charge mass.

**Figure 11:**
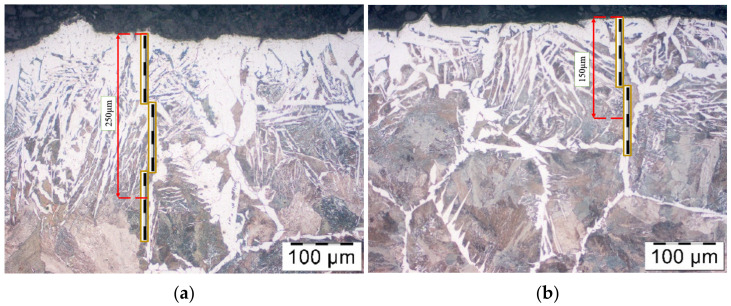
The view of microstructure (decarburization) on the material surface after heating: (**a**) in a manual current process, (**b**) in the newly developed heating system (with the use of an optical microscope Olympus GX51).

Comparing the results obtained in both trials for a heater applied so far and the new elaborated heating technique—for a charge twice as long, due to the production of forgings in a double system—two forgings from one charge (in the old solution, is 53 mm, and in the new, 132 mm with the same diameter and type of charge). We can demonstrate a significant reduction of weight and type of surface scale, which proves a decrease of the negative effect of heating in an oxidizing atmosphere on the charge material. In spite of that, it was necessary to conduct microstructural tests in order to verify the effect on the decarburization of the charge surface. The performed microstructural tests on the surface in both examined cases of induction heating have been presented in Figure 11. During the heating on the manual forging stand, the decarburization in the extreme case equaled 250 µm (Figure 11a). In the case of the developed new heating technique, the decarburization in its maximal point equaled 150 µm, demonstrating much lower values than in the case of the previous solution (Figure 11b).

**Figure 12:**
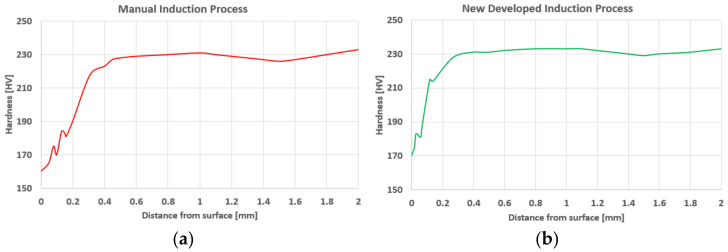
The microhardness test results: (**a**) in a manual current process, (**b**) in the new induction process.

The investigation results point to a reduced effect of holding the material at the forging temperature during heating (above 1000 °C, when rapid oxidation and decarburization takes place), which, in the case of the elaborated solution, affects the quality of the heated charge, and, in consequence, also the end product, the forging. It was confirmed by microhardness tests for both cases (Figure 12).

The performed verification tests showed economic participation of scale in the case of heating with the developed effective heating method, as oxidation on the charge surface was lowered by about 1,1%, and the decarburization of the surface by as much as 40%, in respect to the solution applied so far.

## 4. Conclusions

The study performs a complex analysis and review of the currently applied charge material induction heating methods in hot die forging processes. On this basis, a device for induction heating was designed and then constructed, which made it possible to develop an effective heating method.

The results of simulation tests, which were then confirmed in industrial trials, demonstrated very similar temperature values (scatter 10 °C) both in the core and on the surface, as well as a much shorter time of the charge being held at high temperatures causing strong oxidation. During the industrial 8 h trial, over 5700 charges were heated, among which 59 items were cast away due to underheating at the beginning of the device’s start-up and during production shutdowns, and 6 items due to overheating.

The performed statistical analysis showed a normal temperature distribution with a slight shift towards elevated temperatures. The most occurrences (350–380) were within the scope of 1200 °C to 1206 °C. The reject rate was established at the level of 1.02%, of which 0.1% was due to overheating.

Additionally, the performed shorter 15-min trial (during a continuous work of the device 8 h) also confirmed the correctness of the developed solution. The mean heating temperature was estimated as 1202 ℃, with a maximal deviation of 9 °C and scatter of 19 °C.

The additionally conducted quality examinations showed that the results of the participation of scale was economical in the case of heating by means of the elaborated effective heating method, as oxidation of the charge surface was reduced by about 1,1%, and the surface decarburization—by as much as 40% in respect to the solution applied so far.

It should be emphasized that the developed technology enables work in an automated forging bay owing to the device being equipped with modern automatization systems of charge material preparation.

At present, further research is being performed in the aspect of the expansion of the developed solution with respect to the possibility of heating the charge material with a bigger diameter scope, as well as heating other materials, such as stainless steel, copper, and aluminum alloys. It is assumed that owing to the application of varying frequency inductors and with the proper selection of heating process parameters, such trials should end in success.

## Figures and Tables

**Table 1:** Results of tests with the use of thermocouples.

Sample No.	A [°C]	B [°C]	C [°C]	D [°C]	Lowest Temperature A, B, C [°C]	Temperature Difference on Surface and Core [°C]
1	1173	1173	1171	1176	1171	5
2	1174	1173	1170	1179	1170	9
3	1177	1175	1173	1182	1173	9
4	1173	1172	1170	1177	1170	7
5	1172	1171	1171	1176	1171	5
6	1168	1166	1164	1171	1164	7
7	1180	1181	1178	1185	1178	7
8	1174	1173	1170	1179	1170	9
9	1175	1172	1171	1181	1171	10
10	1170	1168	1165	1175	1165	10

## Data Availability

Not applicable.
